# The devil in more detail: Eco-evolutionary genomics of Tasmanian devil persistence despite range-wide spread of a fatal, transmissible cancer

**DOI:** 10.1371/journal.ppat.1013523

**Published:** 2025-09-24

**Authors:** Andrew Storfer, Marc A. Beer, Dylan Gallinson, Menna E. Jones, Rodrigo Hamede, Hamish McCallum, Mark J. Margres

**Affiliations:** 1 School of Biological Sciences, Washington State University, Pullman, Washington, United States of America; 2 Department of Integrative Biology, University of South Florida, Tampa, Florida, United States of America; 3 School of Natural Sciences, University of Tasmania, Hobart, Tasmania, Australia; 4 Centre for Planetary Health and Food Security, Griffith University, Brisbane, Queensland, Australia; University of Wisconsin-Madison, UNITED STATES OF AMERICA

## Introduction

**Fig 1:**
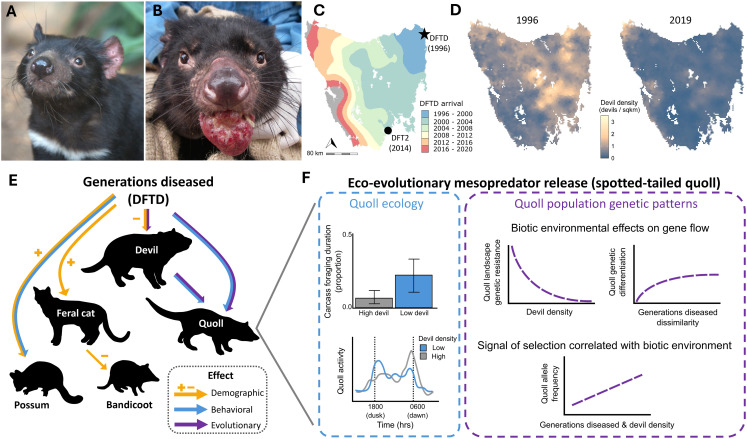
Overview of effects of DFTD on Tasmanian devils from symptoms to population level and community level effects to cascading ecological effects on other native mammals and genetic consequences of ecological relesse of a mesopredator. **A)** Image of a healthy Tasmanian devil adult (Image: Menna Jones). **B)** Image of a Tasmanian devil infected with DFTD (Image: Rodrigo Hamede). **C)** Map of DFTD origin (*), and spread throughout Tasmania; color codes indicate year(s) of introduction to a geographic area. **D)** Devil density prior to DFTD discovery (1996), compared with 2019 (adapted from [2]) demonstrating DFTD-induced population declines. **E)** A trophic cascade, showing the effect of the numbers of generations a devil population is diseased and resulting demographic, behavioral, and evolutionary effects at lower trophic levels. **F)** Evidence for eco-evolutionary shifts in spotted-tailed quolls associated with devils and DFTD. Left: ecological variation among quolls covaries with devil density (adapted from [7,8]); right: quoll population genomic patterns associated with devil density and duration of DFTD’s presence on the landscape, including effects on quoll gene flow and selection (adapted from [11]). Abbreviation: DFTD: devil facial tumor disease.

Emerging infectious diseases (EIDs) pose one of the greatest threats to human and wildlife health. A remarkable example of EIDs are two transmissible cancers that threaten populations of the Tasmanian devil (*Sarcophilis harrisii*), Tasmania’s top predator and scavenger, with possible range-wide extinction (Fig 1A) [1,2]. Devil facial tumor disease (DFTD; Fig 1B) was discovered in 1996 to have originated from a female devil (Fig 1C) is nearly 100% lethal, and has spread from east to west throughout most of the devil’s geographic range [2]. DFT2, a second, independently evolved and male-derived lineage, was discovered in 2016 [3], which is also fatal. DFT2, relative to DFTD, has a limited geographic range, and it has not yet been determined the level of threat it poses for devil persistence. Nonetheless, devils transmit both DFTD/DFT2 via biting during common social interactions, including territoriality, competition for carrion, and male mate guarding of females [4–7]. Indeed, social network analyses show that male devils are more likely to receive potentially DFTD-transmitting bite wounds than females during the mating season [5]. Herein, we provide an update of significant research progress in this study system since our *PLoS Pathogens* Pearl [1] published over 6 years ago; due to the relative paucity of data on DFT2 relative to DFTD, we focus herein on DFTD unless otherwise specifically noted.

Population trajectory modeling of nine DFTD-infected sites showed local devil population declines averaging 77% across Tasmania [2,6] (Fig 1D). The community-level eco-evolutionary effects of devil declines are evident in trophic cascades. Devil declines cause the release of mesopredators, including demographic release of feral cats (*Felis catus*) [8] and changes in timing of foraging behavior of spotted-tailed quolls (*Dasyurus maculatus*) [9,10] (Fig 1E). Increase of feral cat densities results in secondary declines of native mammals, including southern brown bandicoots (*Isodon obesulus*) [8]. Using landscape resistance models, low devil densities (lagged ≥10 quoll generations prior to sampling) correlate with reduced gene flow among spotted-tailed quoll populations [11]. Additionally, generalized dissimilarity models suggest that quoll gene flow is higher among locations with similar devil densities than locations with more distinct devil densities, which may reflect divergent selection or environmentally biased dispersal [11] (Fig 1F).

Initial compartmental epidemiological models of DFTD predicted devil extinction due to predominantly frequency-dependent transmission [4], but more recent studies show evidence of both frequency-dependent and density-dependent transmission [2]. Nonetheless, devils persist throughout their geographic range, and persistence is driven partly by DFTD being a “slow-burning disease” with an extended latent period that often allows infected females to breed in the following mating season, with no evidence of vertical transmission [12]. Next, we summarize ecological phenomena, as well as genomic evidence of devil evolution, DFTD evolution, and devil-DFTD coevolution that collectively help explain the observed persistence of Tasmanian devils.

## Ecological evidence

An individual-based model incorporating individual and temporal variation in pathogen load predicted persistence to 50 generations (i.e., 100 years) of Tasmanian devils via DFTD extirpation as the most probable population outcome (57% of simulations) [12]. Devil extirpation and coexistence of devils and DFTD were less likely (21% and 22%, respectively) [12]. Two empirical studies suggest ecological factors that reduce DFTD transmission in infected populations. As individual tumor load increases, “sickness behavior” reduces a devil’s interactions within its social contact network [13]. Additionally, female home range size and overlap both decrease following DFTD-driven population declines, thereby decreasing opportunities for transmission [14].

## Devil evolution

Demographic history reconstruction analyses using whole-genome data showed repeated declines in effective population sizes of devils throughout the Pleistocene [15] due to repeated recession and declines of glacial ice bridges between mainland Australia and Tasmania and severe El Niño events [16]. Inbreeding was originally hypothesized as a reason for universal devil susceptibility to DFTD [17] and to help justify epidemiological models of extinction and consequent conservation actions [2]. However, despite low genetic variation, multiple studies demonstrate that Tasmanian devils have sufficient standing genetic variation [18–21] to evolve rapidly in response to the extreme selection pressure imposed by DFTD [18–21]. Stahlke and colleagues [21] found evidence for positive selection on 186 candidate loci enriched with genes previously implicated in DFTD-related devil phenotypes. Genes with signatures of historical positive selection did not significantly overlap with those under contemporary selection, suggesting devil evolutionary responses to DFTD are novel.

Expression of innate and adaptive immune-associated genes changes with infection status in Tasmanian devils [22]. That is, in infected devils, adaptive immune genes are generally downregulated by DFTD, and innate immune genes are upregulated [23]. There was no correlation between time since DFTD emergence and immune gene expression pattern [23], but season and/or sex may play a role [23]. These studies were primarily conducted in *in vitro* laboratory experiments. Nonetheless, in field-collected biopsies of infected devils, devil gene expression varies geographically, but there is little evidence for differential expression associated with DFTD infection, suggesting a possible lack of regulatory response to DFTD [24].

## DFTD/DFT2 evolution

Phylogenetic analyses show DFTD, which evolved from a female devil, has diversified into four distinct evolutionary lineages [25]. DFTD and DFT2 are both undifferentiated Schwannomas with similar case fatality rates [3,25]. Phylogenetic analyses suggest DFTD originated between 1982 and 1991 [25,26], whereas DFT2 originated between 2009 and 2012 [25]. DFT2 exhibits more rapid evolutionary rates, including mutation rates, than DFTD; candidate driver mutations also differ between DFTD and DFT2 [25,26]. Pathways showing downregulated gene expression in DFTD relative to devils include DNA damage checkpoints involving *TP53* [24]. A proposed mechanism for DFTD transmission via immune avoidance is upregulation of *ERBB3*, which inhibits *β*_*2*_*m* expression and thereby MHC expression in tumor cells [27]. However, DFT2 still expresses *β*_*2*_*m*, leaving the underlying mechanism of transmission in question [28]. DFTD gene expression, including among cell cycle genes, varies geographically, which may reflect local variation in: 1) relative abundance of tumor lineages; or 2) devil gene expression [24].

Whereas DFTD has spread throughout Tasmania, DFT2 cases have been largely observed inside the d’Entrecasteaux peninsula, but are gradually spreading north, likely due to its more recent origin [3,25,26]*.* DFT2 tumors are significantly more likely to occur on the body of infected animals than DFTD, which is largely restricted to the face [26]. Males are more likely to be infected with DFT2, possibly owing to female recognition of Y chromosome-derived antigens [26]. However, DFTD is equally likely to infect males and females [26]. In cell monocultures, DFT2 grew twice as fast as DFTD but reached lower maximum densities [29]. In co-culture experiments, DFT2 always outcompetes DFTD, even at a 30:70 starting cell ratio [29]. Ongoing studies are testing whether DFT2 outcompetes DFTD in the wild where they co-occur. DFTD appears to be evolving from emergence to endemism.

Phylodynamics analyses of 51 island-wide DFTD genomes show that two of the extant DFTD lineages initially rose in transmission rate, measured by effective reproduction rate (*R*_*E*_), to ~3.5 before declining to *R*_*E*_* *~ 1 at present [30]. Although DFTD has been observed to spread from east to west across Tasmania, phylodynamics analyses suggest that DFTD disperses omnidirectionally [30], resulting in little geographic structure among tumor lineages and the co-occurrence of multiple lineages at single sites [30–32]. Within a single devil population, mtDNA analyses showed that two of the three tumor clades originally present were extirpated within 10–12 years postemergence [31].

Recently, a critique of this work has been published [33], which correctly points out that the genome-wide mutation rate was overestimated in Patton and colleagues [30]. Nonetheless, a re-analysis of the data in Patton and colleagues [30] according to the standards set forth in Stammitz and colleagues [33] *as well as* the data presented from [25] used to justify the critique [30] in Stammnitz and colleagues [33] show that while the over-estimation of the mutation rate results in a slightly later estimated date of origin of DFTD, the main results of [30] remain robust [34], including: 1) omnidirectional spread of DFTD [30,34]; and 2) the decline in *R*_*E*_ from a high of ≅3.5 to ≅1 at present [30,34], fully supporting the original conclusions of evolution to endemism in [30,34].

## Devil-DFTD coevolution

Increasing numbers of cases of spontaneous tumor regression have been found in wild devils [18,35], a phenomenon observed in 1 in 60,000–100,000 human cancer patients. We conducted comparative genomics analyses of eight regressed devils/tumors and seven non-regressed devils/tumors as controls [18,35]. In devils, variation in three genomic regions containing candidate genes related to immune response and cancer risk likely contributes to natural tumor regression. However, there were no non-synonymous substitutions, and identified genetic variants occurred in putative regulatory regions [18]. In tumors, a single point mutation in the 5′ untranslated region of *RASL11a*, a putative tumor suppressor, contributed to tumor regression [35]. *RASL11a* was expressed in regressed DFTD tumors but silenced in non-regressed tumors, consistent with homologous *RASL11a* downregulation in human prostate and colon cancers [35]. Confirming the putative function of this candidate gene, *in vitro* cell culture assays showed that overexpression of *RASL11a* slowed tumor growth relative to wild-type cell lines [35].

**Fig 2:**
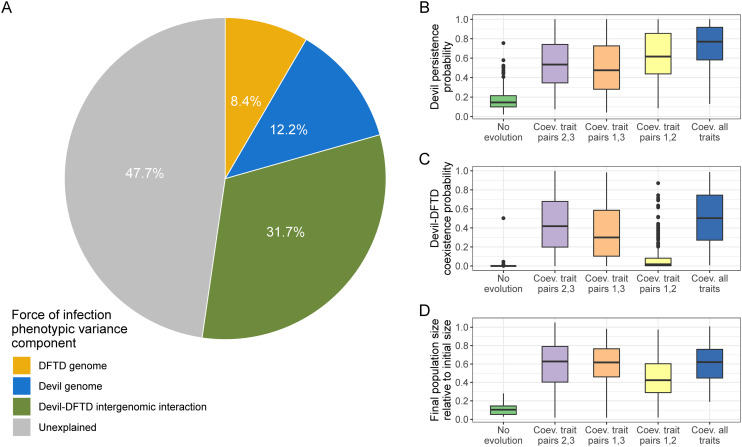
Coevolution in the devil-DFTD system. **A)** Contributions of the devil genome, DFTD genome, and devil × DFTD genomic interactions in explaining variation in how quickly susceptible devils became infected with DFTD (a proxy for force of infection) [32]. Probability of **B)** devil persistence and **C)** devil-DFTD coexistence 50 generations following DFTD arrival. The trait pairs included: (1) devil infection resistance/DFTD transmissibility, (2) devil resistance to tumor growth/DFTD growth rate, and (3) devil tolerance/tumor virulence. **D)** Final devil population size relative to initial population size in simulations where devils and DFTD coexisted for 50 generations (adapted from [33]). Abbreviation: DFTD: devil facial tumor disease.

To test for evidence of coevolution at the genomic level, we used a novel, co-GWAS approach that estimated the contributions of the devil genome, DFTD genome, and devil-DFTD intergenomic interactions to explain variation in how quickly susceptible devils became infected [36]. Remarkably, we found that the proportion of phenotypic variation explained (PVE) by devil-DFTD intergenomic interactions was higher (median PVE = 0.317; 95% CI = 0.293–0.330) than either devil (median PVE = 0.122; 95% CI = 0.108–0.128) or DFTD (median PVE = 0.084; 95% CI = 0.0829–0.0848) genomes alone [36] (Fig 2A). The top interacting variants were significantly enriched for both cancer genes and signatures of selection, providing evidence of a G × G interaction and therefore devil-DFTD coevolution.

Recently, Clement and colleagues [37] published the first individual-based, eco-evolutionary model of devil-DFTD coevolution parameterized with data from nearly two decades of devil demography, DFTD epidemiology, and GWAS. Model simulations showed a higher probability of devil persistence over 50 devil generations than in Wells and colleagues [12] (77% versus 57%; Fig 2B) and a higher likelihood of devil-DFTD coexistence (50% versus 22%; Fig 2C), with greater devil population recovery (60% versus ~50% of pre-disease population sizes; Fig 2D).

## Conclusions and management implications

The evidence presented above strongly supports the contention that DFTD is unlikely to cause Tasmanian devil extinction, although recovery to pre-disease population sizes is unlikely [2,12,37] (Fig 2D). Upon discovery of DFTD, captive insurance devil populations were established in wildlife parks and zoos; captive devils were bred to maximize genetic diversity for potential reintroductions in cases of localized extirpations or species-wide extinction. More recently, an insurance population of devils was established on Maria Island off the coast of Tasmania with no history of devils or DFTD. However, while internal relatedness (a measure of inbreeding) has decreased in the zoo and park populations due to active breeding management, it has not in the Maria Island population, where breeding remains unmanaged [38]. Translocations of Tasmanian devils from this island for demographic or genetic rescue of mainland populations remain controversial [39]. Not only can introducing evolutionarily naïve devils into populations that have experienced selection by DFTD disrupt local adaptation, but increasing densities of infected populations can fuel the epizootic [2,31,39, but see 40]. Nonetheless, Farquharson and colleagues [41] suggest that putatively functional genetic diversity of wild devil populations is largely represented in the insurance metapopulation.

A recent population viability analysis suggests maintenance of captive breeding populations and reintroductions into depauperate wild populations is extremely costly and likely to provide little demographic benefit [42]. However, successful development of a vaccine would be the most promising and cost-effective avenue for devil maintenance or recovery [42]. Whilst progress towards vaccine development continues, whether this will be possible remains unclear. Nonetheless, management interventions may not be needed as the studies we summarize herein, taken together, provide strong evidence that devils and DFTD are coevolving naturally. The extinction threat of DFT2 remains to be determined.
